# Checkpoint Travel Numbers as a Proxy Variable in Population-Based Studies During the COVID-19 Pandemic: Validation Study

**DOI:** 10.2196/44950

**Published:** 2023-08-29

**Authors:** Jennifer M Kreslake, Kathleen Aarvig, Hope Muller-Tabanera, Donna M Vallone, Elizabeth C Hair

**Affiliations:** 1 Schroeder Institute Truth Initiative Washington, DC United States; 2 Department of Health, Behavior and Society Johnks Hopkins Bloomberg School of Public Health Baltimore, MD United States; 3 College of Global Public Health New York University New York, NY United States

**Keywords:** research methods, public health, data quality, psychosocial factors, history, COVID-19, social, behavioral, validation, social distancing, tracking survey, survey, pandemic

## Abstract

**Background:**

The COVID-19 pandemic had wide-ranging systemic impacts, with implications for social and behavioral factors in human health. The pandemic may introduce history bias in population-level research studies of other health topics during the COVID-19 period.

**Objective:**

We sought to identify and validate an accessible, flexible measure to serve as a covariate in research spanning the COVID-19 pandemic period.

**Methods:**

Transportation Security Administration checkpoint travel numbers were used to calculate a weekly sum of daily passengers and validated against two measures with strong face validity: (1) a self-reported item on social distancing practices drawn from a continuous tracking survey among a national sample of youths and young adults (15-24 years) in the United States (N=45,080, approximately 280 unique respondents each week); and (2) Google’s Community Mobility Reports, which calculate daily values at the national level to represent rates of change in visits and length of stays to public spaces. For the self-reported survey data, an aggregated week-level variable was calculated as the proportion of respondents who did not practice social distancing that week (January 1, 2019, to May 31, 2022). For the community mobility data, a weekly estimate of change was calculated using daily values compared to a 5-week prepandemic baseline period (January 3, 2020, to February 6, 2020). Spearman rank correlation coefficients were calculated for each comparison.

**Results:**

Checkpoint travel data ranged from 668,719 travelers in the week of April 8, 2020, to nearly 15.5 million travelers in the week of May 18, 2022. The weekly proportion of survey respondents who did not practice social distancing ranged from 18.1% (n=42; week of April 15, 2020) to 70.9% (n=213; week of May 25, 2022). The measures were strongly correlated from January 2019 to May 2022 (ρ=0.90, *P*<.001) and March 2020 to May 2022 (ρ=0.87, *P*<.001). Strong correlations were observed when analyses were restricted to age groups (15-17 years: ρ=0.90; *P*<.001; 18-20 years: ρ=0.87; *P*<.001; 21-24 years: ρ=0.88; *P*<.001), racial or ethnic minorities (ρ=0.86, *P*<.001), and respondents with lower socioeconomic status (ρ=0.88, *P*<.001). There were also strong correlations between the weekly change from the baseline period for checkpoint travel data and community mobility data for transit stations (ρ=0.92, *P*<.001) and retail and recreation (ρ=0.89, *P*<.001), and moderate significant correlations for grocery and pharmacy (ρ=0.68, *P*<.001) and parks (ρ=0.62, *P*<.001). A strong negative correlation was observed for places of residence (ρ=−0.78, *P*<.001), and a weak but significant positive correlation was found for workplaces (ρ=0.24, *P*<.001).

**Conclusions:**

The Transportation Security Administration’s travel checkpoint data provide a publicly available flexible time-varying metric to control for history bias introduced by the pandemic in research studies spanning the COVID-19 period in the United States.

## Introduction

On March 11, 2020, the outbreak of the disease resulting from SARS-CoV-2 (COVID-19) was declared a pandemic by the World Health Organization [1]. As cases surged in the United States, public health and government officials engaged in unprecedented efforts to limit the spread of the virus. Without effective pharmacological interventions and vaccines, social distancing emerged as the primary mitigation strategy [2,3]. Furthermore, many governments enacted stay-at-home mandates and the closing of nonessential businesses, with the reasoning that with fewer people traveling outside their homes, there would be less close physical contact between infected and uninfected individuals [4]. According to a growing body of research evaluating the effectiveness of these interventions, these actions were instrumental in reducing infection rates [5].

The COVID-19 pandemic, and subsequent control measures, had wide-ranging systemic impacts with implications for social and behavioral factors in human health. The time-dependent nature of the pandemic introduces history bias into longitudinal and time series analyses, with the pandemic’s many impacts acting as confounders in population-level research studies. A commentary by Tuttle et al [6] noted that potential bias introduced by the COVID-19 pandemic has important considerations for analysis and interpretation in clinical research, and suggested that potential confounding may be addressed by examining pre-and postpandemic data with a control for COVID-19 behaviors. Such a control variable should have validity within a variety of target populations addressed by public health (eg, by age, socioeconomic status, race, or ethnicity).

Mobility data have the ability to provide nearly real-time information about changes in patterns of human movement as measured by activity on mobile phones, GPS tracking, and social media platforms. For the past several years, researchers have used mobility data to inform epidemiological modeling, situational awareness, and resource allocation during public health crises [7-14]. Thus, most of the available literature leverages mobility data to track the movements of individuals during the COVID-19 pandemic, to identify areas of high transmission, or evaluate the efficacy of interventions, such as social distancing [7-12]. In contrast, few studies have applied these data to other areas of public health research concurrent with the COVID-19 pandemic. It was 1 study from the United Kingdom that used mobility data to assess the impact of COVID-19 pandemic restrictions on physical activity levels [14]. Another in the United States established an association between county-level social distancing patterns during the COVID-19 pandemic and other county-level indicators of health behavior (eg, smoking, obesity, flu vaccination, and mammography screening) using data from GPS-enabled mobile devices [13].

The purpose of this study is to identify a suitable control variable for longitudinal or time series social and behavioral studies that span the COVID-19 pandemic period. We sought to determine whether air travel metrics can be used as a time-varying endogenous indicator of COVID-19 impacts on societal activities. The airline industry was detrimentally impacted by the COVID-19 pandemic. Passenger air travel was the largest contributor to industry losses during the pandemic, when the number of air travel passengers dropped by approximately 60% in 2020 [15,16]. Thus, we believe this may be a suitable control variable to integrate retrospectively into data sets that span the COVID-19 period. We will validate this measure against 2 time-varying population-level measures with strong face validity: the first is calculated by aggregating individual-level self-reported social distancing practices at a weekly level, drawn from a continuous tracking survey of youths and young adults in the United States; and the second is from Google’s COVID-19 Community Mobility Reports, which calculate daily values at the national level to represent rates of change in visits and length of stays to public spaces [9].

## Methods

### Data and Measures

#### Checkpoint Travel Data

Publicly available data from the Transportation Security Administration (TSA) was used to construct an exogenous indicator of COVID-19’s population-level impacts. TSA maintains a daily count of travelers screened at TSA airport checkpoints. As of this writing, data have been made available by TSA on the internet from 2019 to the present [17]. A weekly checkpoint travel variable was constructed by summing the TSA’s daily checkpoint travel numbers within each week (Wednesday-Tuesday), which was used for the analysis correlating this measure to self-reported social distancing (Multimedia Appendix 1). For analyses comparing checkpoint travel data to community mobility data, the weekly checkpoint travel data were calculated as a weekly change score compared to the median checkpoint travel value for the corresponding day of the week, during the 5-week prepandemic period spanning January 3, 2020, to February 6, 2020.

#### Self-Reported Social Distancing Data

The self-reported measure of social distancing used data from a cross-sectional, continuous tracking survey of youths and young adults (aged 15-24 years) drawn from the Dynata national web-based panel. The survey is administered continuously (ie, sampling occurs every day) and receives responses from approximately 280 unique participants per week. Participants who completed a survey between January 1, 2019, to May 31, 2022 (N=45,080) were included in the analysis. Sampling quotas were used and survey weights were applied using age, race or ethnicity, and sex based on demographic benchmarks from the US Census to approximate a national sample.

A self-reported, single-response item on the frequency of social distancing during the past week was added to the survey instrument on March 25, 2020, and remained throughout the study period. Respondents were presented with the following question: “Social distancing means people stay home as much as possible to prevent catching and spreading the virus, and if they go outside, they stay at least 6 feet away at all times from any person who they don’t live with. Other terms for social distancing that some people use are self-quarantine and self-isolation. In the past week, have you done social distancing…?” Responses were coded as 0=at all times, 1=usually, 2=sometimes, 3=very little, and 4=not at all. Responses to the self-reported measure on social distancing were dichotomized (0=at all times or usually, 1=sometimes or very little or not at all). An aggregated week-level variable was calculated as the proportion of respondents that week who reported social distancing sometimes or very little or not at all (ie, coded as “1” for the dichotomized variable; Multimedia Appendix 2). In weeks prior to March 9, 2020, respondents were coded as “1” to reflect a lack of social distancing during the pre–COVID-19 period. The survey did not ask about social distancing during the first 2 weeks of the pandemic (weeks of March 9 and March 16), so the Next Observation Carried Backward method was used to impute values for the weeks of March 9 and March 16 using the proportions from the week of March 23. The aggregated values in weeks prior to March 9 were 100%, representing no social distancing across all respondents (n=280) within each of those weeks.

We constructed 2 additional aggregate measures of self-reported social distancing using only data from population subgroups (ie, racial or ethnic minorities and lower socioeconomic status) to determine if the correlation to the endogenous measure is similar for target populations commonly found in public health research. For racial or ethnic minorities, we limited the aggregation of the self-reported measure to those from respondents who self-identified as being non-White (ie, Black or African American, Asian, Native Hawaiian or other Pacific Islander, American Indian or Alaska Native, or multiracial) or Hispanic (n=14,657). For socioeconomic status, we used a subjective measure of household income that has been validated among young adults. Young adult respondents are asked: “Considering your own income and the income from any people who help you, how would you describe your own overall financial situation? Would you say you… (live comfortably; meet needs with a little left over; just meet basic expenses with nothing left over; don’t meet basic expenses).” This measure was adapted to respondents aged 15 to 17 years, who are asked: “Considering all income earners in your family, how would you describe your family’s overall financial situation, would you say you…” with the same response options as the original measure. The aggregated measure for low socioeconomic status was limited to respondents who reported having their basic needs met with nothing left over or not having their basic needs met (n=30,054).

#### COVID-19 Community Mobility Data

Google’s COVID-19 Community Mobility Data compare how visits and length of stay in various sites within communities changed compared to a 5-week prepandemic baseline period (January 3, 2020, to February 6, 2020) [18]. Data are drawn from mobile devices where users are signed into their Google accounts. Data collection is limited to Google users who have elected to activate their Location History; the default setting is off. To be comparable to the TSA’s checkpoint travel data, the analysis for this paper used country-level data for the United States.

A daily change score is calculated using values from each day compared to the median value for the corresponding day of the week during the 5-week baseline period. For the purposes of this study, we calculated weekly change scores for the TSA data from the same baseline period.

Google classifies community locales using the following categories: retail and recreation (eg, restaurants and cafes, shopping centers, theme parks, museums, libraries, and movie theaters), grocery and pharmacies (eg, grocery markets, food warehouses, farmer’s markets, specialty food shops, drug stores, and pharmacies), parks (eg, local parks, national parks, public beaches, marinas, dog parks, plazas, and public gardens), transit stations (eg, subway, bus, and train stations), workplaces, and places of residence.

### Ethics Approval

The study was conducted in accordance with the Declaration of Helsinki. Checkpoint travel data and community mobility report data were obtained from anonymous, aggregated publicly available sources (TSA and Google, respectively). The protocol for the survey providing self-reported social distancing data was approved by the Advarra Institutional Review Board (Protocol ID: Pro0010120). Informed consent was obtained from all survey respondents aged 18 years or older, or from a parent or legal guardian for all respondents under 18 years of age. Respondent incentives were in accordance with their membership in a web-based panel maintained by Dynata.

### Statistical Analysis

The Shapiro-Wilk test for normality determined that measures were not normally distributed for weekly air travel (W=0.919, *P*<.001), aggregated weekly social distancing (W=0.942, *P*<.001), and community mobility data (grocery: W=0.892, *P*<.001; recreation: W=0.877, *P*<.001; transit: W=0.986, *P*<.001) during the pandemic period, likely due to the high level of social distancing and reduction in travel observed during a short duration at the beginning of the pandemic period.

Spearman rank correlation coefficient was calculated for the weekly sum of travel checkpoint data, as were (1) the weekly proportion of survey respondents who did not practice social distancing and (2) the weekly percent change in visits to public spaces compared to the prepandemic baseline.

A secondary analysis was conducted using first-order differencing to remove the effects of the overall trend spanning the study period and determine the checkpoint travel data’s usage in controlling for weekly change.

All analyses were conducted using RStudio (version 2021.09.0), developed by Posit, formerly known as RStudio Inc.

## Results

### Correlations Between Checkpoint Travel Data and Self-Reported Social Distancing

**Figure 1:**
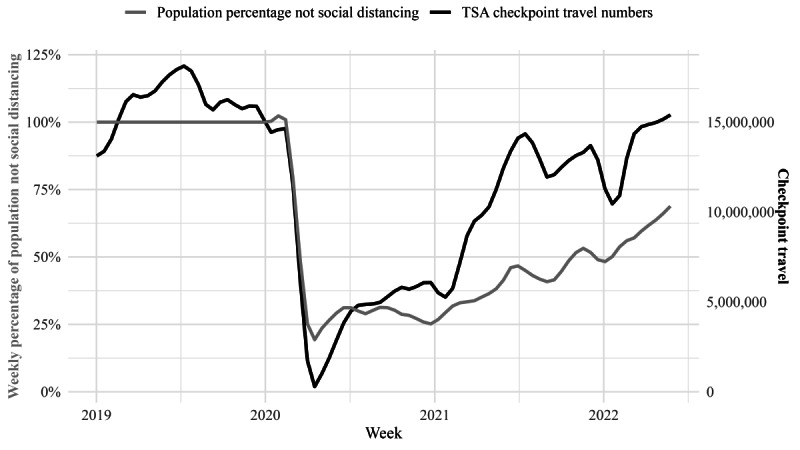
Weekly checkpoint travel volume and proportion of youths and young adults reporting not social distancing before and during the COVID-19 pandemic. TSA: Transportation Security Administration.

The relationship between the checkpoint travel data and self-reported social distancing measure (Figure 1) was visually inspected. The plot suggested a positive linear relationship between checkpoint travel numbers with no significant outliers when examining the prepandemic period and pandemic periods separately. Additional graphs were created to inspect whether a positive relationship could also be observed between checkpoint travel data and population subgroups (Multimedia Appendix 3) and whether checkpoint travel varied in relation to surges in COVID-19 variants (Multimedia Appendix 4).

Checkpoint travel data showed the minimum weekly sum of travelers (n=668,719) occurring the week of April 8, 2020, peaking at nearly 15.5 million the week of May 18, 2022 (Multimedia Appendix 1). The weekly proportion of survey respondents who did not practice social distancing followed a similar trend, ranging from 18.1% (n=42; week of April 15, 2020) to 70.9% (n=213; week of May 25, 2022; Multimedia Appendix 2). The measures were strongly and significantly correlated from January 2019 to May 2022 and March 2020 to May 2022 (Table 1). Strong correlations were observed when analyses were restricted to age groups (ie, 15-17, 18-20, and 21-24 years), racial or ethnic minorities, and respondents with lower socioeconomic status, all of which were significant (Table 1). First-order differencing correlations between weekly checkpoint travel data and self-reported social distancing were not significant for either the overall sample, neither for racial or ethnic minorities nor for respondents with lower socioeconomic status. A weak positive and significant correlation was observed for first-order differences of weekly checkpoint travel data and self-reported social distancing among youths aged 15-17 years (Table 1), but no other correlations using first-order differencing were significant for other age groups.

**Table 1:** Correlation of Transportation Security Administration’s weekly checkpoint travel volume with other weekly indicators of behavioral impacts of the COVID-19 pandemic, January 2019 to May 2022.

		ρ		*P* value
**Self-reported social distancing^a^**				
	Total population (aged 15-24 years)	0.90	<.001	
	Racial or ethnic minorities	0.86	<.001	
	Low SES^b^	0.88	<.001	
	Age 15-17 years	0.90	<.001	
	Age 18-20 years	0.87	<.001	
	Age 21-24 years	0.88	<.001	
**Change in community mobility^c^**				
	Transit stations	0.92	<.001	
	Retail and recreation	0.89	<.001	
	Grocery and pharmacy	0.68	<.001	
	Parks	0.62	<.001	
	Workplaces	0.24	<.001	
	Places of residence	−0.78	<.001	

^a^Aggregated weekly percent reporting socially distancing sometimes or very little or not at all in the past week.

^b^SES: socioeconomic status.

^c^Aggregated weekly change score compared to baseline weeks (January 3, 2020, to February 6, 2020).

A consistent visual pattern was observed when we inspected checkpoint travel data during the 10-week period before and after the emergence of new variants or surge in cases over the course of the study period (Multimedia Appendix 3). New variants included Alpha B.1.1.7 (emerged December 29, 2020), Delta B.1.617.2 (emerged June 1, 2021, and surged July 27, 2021), and Omicron B.1.1.529 (emerged December 1, 2021), with surges of both Omicron and Delta variants identified on January 1, 2022 [19]. Increased checkpoint travel numbers reflected a rise in air travel during the winter holiday and summer months. A rise in checkpoint travel numbers directly preceded surges in new variants of COVID-19: Alpha B.1.1.7 (December 2020), Delta B.1.617.2 (July 2021), and Omicron B.1.1.529 and Delta B.1.617.2 (December 2021). A drop in checkpoint travel numbers immediately followed each surge before rising again in subsequent months.

### Correlations Between Checkpoint Travel Data and Community Mobility Data

**Figure 2:**
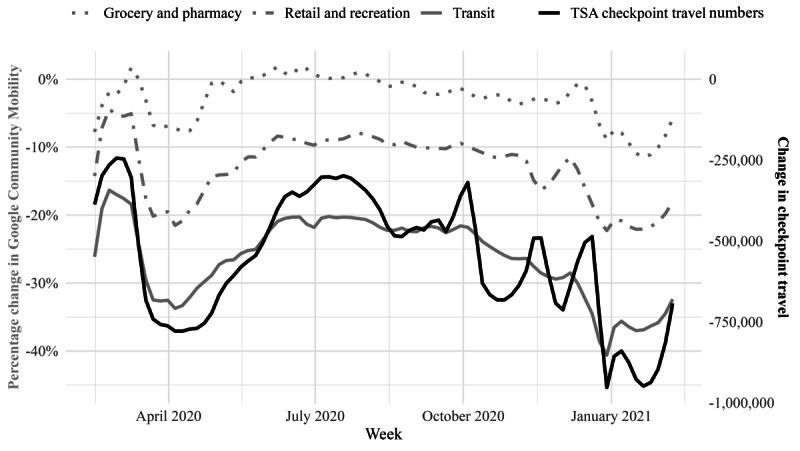
Change in weekly checkpoint travel volume and community mobility estimates compared to baseline weeks preceding the COVID-19 pandemic (January 3, 2020, to February 6, 2020). TSA: Transportation Security Administration.

The relationship between the checkpoint travel measure and community mobility measure was visually inspected (Figure 2).

There was a strong positive, significant correlation between the weekly change from the baseline period for checkpoint travel data and community mobility data for transit stations and retail and recreation, and moderate significant correlations for grocery and pharmacy as well as parks (Table 1). A strong negative correlation was observed for places of residence, and a weak but significant positive correlation was found for workplaces (Table 1).

First-order differencing of changes in weekly checkpoint travel and community mobility from baseline had moderate positive and significant correlations for transit stations, retail and recreation, grocery and pharmacy, and parks. A weak but significant negative correlation was observed for places of residence and workplaces (Table 1).

## Discussion

### Principal Findings

The TSA’s daily travel checkpoint data provide a publicly available, flexible metric that may be easily incorporated into national data sets to serve as a proxy for the intensity of COVID-19 activity in the population at a given time interval. As of this writing, these data are available on the internet and span back to 2019. It is unknown whether these data will continue to be tracked and published on the internet indefinitely.

Future data collection efforts may consider including a self-reported measure on social distancing in population-based surveys for as long as new strains of COVID-19 are expected to impact social dynamics to a varying extent over time. Public health officials have noted that society continues to be vulnerable to other emergent and resurgent diseases with pandemic potential [20]. This measure may be applicable to other pandemic diseases requiring self-isolation and quarantine at a broad scale.

Changes in checkpoint travel data used in our study correlated in the expected direction with changes in community mobility, with strong correlations observed for transit stations, and retail and recreation sites. A moderate correlation was observed with places to access essential goods and services (ie, grocery stores and pharmacies) as well as parks, which were popular outdoor recreational options during the pandemic. A weak correlation was observed for workplaces, likely reflecting the adoption of remote work policies by many employers. As expected, a strong negative correlation was observed between checkpoint travel data and being at home. Google ceased publication of its COVID-19 Community Mobility Reports (used in this study) as of October 15, 2022, yet daily checkpoint travel data remain available. Mobility data are subject to proprietary data processing methods and policies, including changes in parameters used by Google in the change score calculation over time (for example, to account for shifts in regional population size or retailer density). For this reason, Google cautions against comparing time periods that span longer than 6 months. By contrast, checkpoint travel data do not rely on third parties to generate estimates and can be applied to data sets spanning multiple years. Checkpoint travel data are available in the years preceding the COVID-19 pandemic, whereas the mobility data used in our study could only be viewed as a percent change from the prepandemic baseline determined by the company.

First-order differencing analysis at a weekly level found few significant correlations between checkpoint travel data and self-reported social distancing, and weak to moderate correlations with community mobility data. These results suggest that the measure is less useful as a control for time intervals with high granularity, such as time series studies examining week-to-week changes within the COVID-19 period. Similarly, Google cautions against using its Community Mobility Reports to compare day-to-day activity and calculates its change score from a median value from a pre-COVID baseline period spanning several weeks. The use of checkpoint travel data as a proxy for COVID-19 activity is, therefore, best applied to analyses that need to control for time-dependent trends attributable to the COVID-19 pandemic, such as analyses that encompass the prepandemic and pandemic periods, or the first several months of the pandemic when lockdowns occurred compared to periods later in the pandemic after societal activities began to resume.

### Limitations

This study was subject to limitations. First, the daily air travel metrics of interest to this study were validated against a national sample of youths and young adults, rather than a nationally representative data set of the general population. While we are encouraged that the strength of the correlation was similar within defined subgroups in our sample, these subgroups remain restricted to people younger than 25 years. It is unclear how similar the social distancing practices of young people in our sample would be to those of the general adult population.

Second, the metric against which checkpoint travel data were validated (Google Community Mobility Data) is only available for Google account users with Location History settings activated and thus is also subject to an unknown degree and direction of bias.

Finally, while initially checkpoint travel numbers declined sharply following the emergence of COVID-19 and society’s attempts to limit its transmission, later in the pandemic a predictive pattern was evident between a rise in checkpoint travel numbers and subsequent COVID-19 variant surges. A rise in seasonal travel (ie, holidays and summer vacations) appeared to prompt surges in the incidence of emerging COVID-19 variants. The steep drop in checkpoint travel numbers immediately after each COVID-19 surge may have been driven in part by individuals’ desire to avoid further transmission, but it is also likely that these are normal seasonal fluctuations after a popular travel period. These data illustrate the extent to which air travel not only reflects but almost certainly contributes to COVID-19 transmission. Therefore, although they may serve as an appropriate proxy for COVID-19 dynamics in the population, checkpoint travel numbers are not a completely independent indicator of this phenomenon.

### Conclusions

We recommend using the TSA’s checkpoint travel data in longitudinal or time series studies that span the COVID-19 pandemic period. This variable can control for the potential confounding effect of COVID-19 activity in population-based studies of a range of public health topics and within demographic subgroups.

## Appendix

Multimedia Appendix 1 Weekly sum of travelers screened at Transportation Security Administration (TSA) checkpoints, January 2019-May 2022.

Multimedia Appendix 2 Proportion of youth and young adults reporting social distancing during the past week (approximately 280 unique respondents per week, N=45,080), March 2020-May 2022.

Multimedia Appendix 3 Weekly percent of subgroups of respondents not social distancing: (A) age (15-17 years, 18-20 years, 21-24 years), (B) lower socioeconomic status, and (C) racial/ethnic minorities.

Multimedia Appendix 4 Weekly checkpoint travel volume and the emergence of new COVID-19 variants, 10 weeks before and after surges of (A) Alpha B.1.1.7, (B) Delta B.1.617.2, and (C) Omicron B.1.1.529 and Delta B.1.617.2.

## Abbreviations

TSA: Transportation Security Administration

## Data Availability

The Transportation Security Administration’s checkpoint travel data and Google’s COVID-19 Community Mobility Reports are publicly available [1,2]. Investigators seeking access to the self-reported social distancing survey data used in this study should make a written request to Truth Initiative authors and submit a detailed research plan including the purpose of the proposed research, required variables, duration of the analysis phase, institutional review board approval with Federalwide Assurance information, and investigator training in human subjects. A data sharing agreement is required for use of all data. Truth Initiative does not share data with tobacco industry representatives or affiliated researchers. Approved investigators must access data sets via an analytic Portal owned and administered by Truth Initiative.
