# Supplementary material for: Checkpoint Travel Numbers as a Proxy Variable in Population-Based Studies During the COVID-19 Pandemic: Validation Study
Source: JMIR Public Health Surveill. 2023 Aug 29;9:e44950. doi: 10.2196/44950 (PMC10467631; doi:10.2196/44950)
Supplement: Multimedia Appendix 1 [file publichealth_v9i1e44950_app1.docx]

| **Week**  **(Start Date)** | **Weekly sum of travelers** |
| --- | --- |
| 1/2/2019 | 14,075,444 |
| 1/17/2019 | 12,768,712 |
| 1/17/2019 | 13,535,152 |
| 1/23/2019 | 12,616,096 |
| 1/30/2019 | 12,814,756 |
| 2/6/2019 | 13,579,859 |
| 2/13/2019 | 15,326,240 |
| 2/20/2019 | 15,256,253 |
| 2/27/2019 | 15,791,133 |
| 3/6/2019 | 16,587,096 |
| 3/13/2019 | 16,809,984 |
| 3/20/2019 | 16,601,203 |
| 3/27/2019 | 15,948,328 |
| 4/3/2019 | 16,304,391 |
| 4/10/2019 | 16,518,946 |
| 4/17/2019 | 16,471,979 |
| 4/24/2019 | 16,291,857 |
| 5/4/2019 | 16,504,741 |
| 5/8/2019 | 16,833,480 |
| 5/17/2019 | 17,611,948 |
| 5/22/2019 | 16,479,200 |
| 5/29/2019 | 17,193,328 |
| 6/6/2019 | 17,886,586 |
| 6/12/2019 | 18,181,268 |
| 6/19/2019 | 18,357,538 |
| 6/26/2019 | 16,625,081 |
| 7/3/2019 | 18,237,716 |
| 7/10/2019 | 18,060,538 |
| 7/17/2019 | 18,258,668 |
| 7/24/2019 | 18,134,144 |
| 7/31/2019 | 17,926,208 |
| 8/7/2019 | 17,434,968 |
| 8/14/2019 | 16,980,084 |
| 8/22/2019 | 16,313,845 |
| 8/29/2019 | 14,356,386 |
| 9/4/2019 | 15,301,063 |
| 9/12/2019 | 15,958,029 |
| 9/18/2019 | 16,167,879 |
| 9/25/2019 | 15,843,329 |
| 10/3/2019 | 16,407,640 |
| 10/9/2019 | 17,096,892 |
| 10/16/2019 | 16,678,548 |
| 10/23/2019 | 15,102,113 |
| 10/30/2019 | 15,904,308 |
| 11/6/2019 | 15,754,887 |
| 11/13/2019 | 15,453,847 |
| 11/20/2019 | 15,388,740 |
| 11/27/2019 | 16,998,520 |
| 12/4/2019 | 14,942,048 |
| 12/11/2019 | 15,767,487 |
| 12/18/2019 | 16,551,331 |
| 12/26/2019 | 16,394,363 |
| 1/2/2020 | 14,540,005 |
| 1/9/2020 | 14,021,879 |
| 1/15/2020 | 13,998,136 |
| 1/22/2020 | 13,451,510 |
| 1/29/2020 | 13,622,832 |
| 2/5/2020 | 14,935,213 |
| 2/12/2020 | 15,773,989 |
| 2/19/2020 | 15,401,003 |
| 2/26/2020 | 12,136,296 |
| 3/4/2020 | 13,180,920 |
| 3/12/2020 | 8,331,153 |
| 3/21/2020 | 2,649,356 |
| 3/26/2020 | 1,124,145 |
| 4/1/2020 | 774,555 |
| 4/9/2020 | 668,719 |
| 4/15/2020 | 711,801 |
| 4/22/2020 | 871,889 |
| 4/29/2020 | 1,101,643 |
| 5/6/2020 | 1,376,284 |
| 5/13/2020 | 1,681,083 |
| 5/20/2020 | 2,057,872 |
| 5/27/2020 | 2,142,128 |
| 6/3/2020 | 2,761,593 |
| 6/10/2020 | 3,396,959 |
| 6/17/2020 | 3,835,794 |
| 6/24/2020 | 4,188,533 |
| 7/1/2020 | 4,712,355 |
| 7/8/2020 | 4,659,144 |
| 7/16/2020 | 4,617,320 |
| 7/22/2020 | 4,639,816 |
| 7/29/2020 | 4,871,099 |
| 8/5/2020 | 4,933,177 |
| 8/12/2020 | 5,024,392 |
| 8/19/2020 | 4,794,493 |
| 8/26/2020 | 4,664,739 |
| 9/2/2020 | 5,456,947 |
| 9/9/2020 | 4,739,745 |
| 9/16/2020 | 5,011,906 |
| 9/23/2020 | 5,185,466 |
| 9/30/2020 | 5,367,789 |
| 10/7/2020 | 6,016,836 |
| 10/14/2020 | 6,020,983 |
| 10/21/2020 | 5,846,064 |
| 10/28/2020 | 5,379,416 |
| 11/4/2020 | 5,532,875 |
| 11/11/2020 | 5,621,704 |
| 11/18/2020 | 6,859,882 |
| 11/25/2020 | 5,916,573 |
| 12/2/2020 | 4,727,999 |
| 12/9/2020 | 5,015,631 |
| 12/16/2020 | 7,189,935 |
| 12/23/2020 | 7,171,155 |
| 12/30/2020 | 6,713,361 |
| 1/6/2021 | 4,935,880 |
| 1/13/2021 | 5,188,395 |
| 1/20/2021 | 4,633,226 |
| 1/27/2021 | 4,742,716 |
| 2/3/2021 | 5,424,687 |
| 2/10/2021 | 6,513,028 |
| 2/17/2021 | 6,512,227 |
| 2/24/2021 | 6,876,889 |
| 3/3/2021 | 7,466,500 |
| 3/10/2021 | 8,775,865 |
| 3/17/2021 | 9,409,074 |
| 3/24/2021 | 9,777,193 |
| 3/31/2021 | 10,072,660 |
| 4/7/2021 | 9,706,451 |
| 4/14/2021 | 9,468,893 |
| 4/21/2021 | 9,492,921 |
| 4/28/2021 | 9,914,444 |
| 5/5/2021 | 10,882,658 |
| 5/12/2021 | 11,402,521 |
| 5/19/2021 | 11,799,032 |
| 5/26/2021 | 12,336,065 |
| 6/2/2021 | 12,338,533 |
| 6/9/2021 | 13,063,559 |
| 6/16/2021 | 13,721,770 |
| 6/23/2021 | 13,985,595 |
| 6/30/2021 | 13,911,135 |
| 7/7/2021 | 14,167,658 |
| 7/14/2021 | 14,473,913 |
| 7/21/2021 | 14,298,363 |
| 7/28/2021 | 14,324,951 |
| 8/4/2021 | 13,871,948 |
| 8/11/2021 | 13,385,784 |
| 8/18/2021 | 12,552,921 |
| 8/27/2021 | 11,606,130 |
| 9/1/2021 | 12,357,271 |
| 9/8/2021 | 11,192,971 |
| 9/15/2021 | 11,959,650 |
| 9/22/2021 | 12,196,862 |
| 9/29/2021 | 12,261,291 |
| 10/6/2021 | 13,307,744 |
| 10/13/2021 | 13,126,107 |
| 10/20/2021 | 13,029,349 |
| 10/27/2021 | 12,301,325 |
| 11/3/2021 | 12,594,029 |
| 11/10/2021 | 12,970,131 |
| 11/17/2021 | 14,419,320 |
| 11/24/2021 | 14,180,230 |
| 12/1/2021 | 12,427,698 |
| 12/8/2021 | 12,759,663 |
| 12/15/2021 | 14,290,683 |
| 12/22/2021 | 13,667,575 |
| 12/30/2021 | 12,941,175 |
| 1/5/2022 | 10,248,211 |
| 1/12/2022 | 10,285,478 |
| 1/19/2022 | 9,534,456 |
| 1/26/2022 | 9,909,642 |
| 2/2/2022 | 10,119,615 |
| 2/10/2022 | 11,644,434 |
| 2/16/2022 | 13,765,716 |
| 2/23/2022 | 13,433,214 |
| 3/2/2022 | 13,582,539 |
| 3/9/2022 | 14,664,325 |
| 3/16/2022 | 15,004,851 |
| 3/23/2022 | 14,671,576 |
| 3/30/2022 | 14,461,054 |
| 4/6/2022 | 14,818,676 |
| 4/13/2022 | 15,066,512 |
| 4/20/2022 | 14,766,919 |
| 4/28/2022 | 14,572,188 |
| 5/4/2022 | 14,717,712 |
| 5/11/2022 | 15,395,150 |
| 5/18/2022 | 15,481,889 |
| 5/25/2022 | 15,423,167 |
