# Supplementary material for: Checkpoint Travel Numbers as a Proxy Variable in Population-Based Studies During the COVID-19 Pandemic: Validation Study
Source: JMIR Public Health Surveill. 2023 Aug 29;9:e44950. doi: 10.2196/44950 (PMC10467631; doi:10.2196/44950)
Supplement: Multimedia Appendix 2 [file publichealth_v9i1e44950_app2.docx]

|  | **% reporting social distancing in the past week…** | | | | |
| --- | --- | --- | --- | --- | --- |
| **Week  (Start Date)** | **At all times** | **Usually** | **Sometimes** | **Very Little** | **Not at all** |
| 3/26/2020 | 44.5 | 25.8 | 19.6 | 4.8 | 5.4 |
| 4/1/2020 | 53.4 | 19.9 | 16.6 | 4.6 | 5.5 |
| 4/9/2020 | 52.1 | 29.1 | 11.8 | 4.2 | 2.8 |
| 4/15/2020 | 52.1 | 29.8 | 12.7 | 2.7 | 2.7 |
| 4/22/2020 | 50.7 | 25.3 | 18.4 | 2.1 | 3.5 |
| 4/29/2020 | 48.2 | 24.9 | 20.7 | 4.1 | 2.1 |
| 5/6/2020 | 50.4 | 23.6 | 21.4 | 2.5 | 2 |
| 5/13/2020 | 44.9 | 30.7 | 20 | 1.3 | 3.1 |
| 5/20/2020 | 42.1 | 32.1 | 20.7 | 3.7 | 1.4 |
| 5/27/2020 | 36.3 | 34.7 | 20.5 | 5.1 | 3.4 |
| 6/3/2020 | 36.6 | 36.4 | 17.4 | 5.9 | 3.8 |
| 6/10/2020 | 38.5 | 32.8 | 18.6 | 5.5 | 4.6 |
| 6/17/2020 | 27.5 | 39.6 | 20.4 | 7.3 | 5.2 |
| 6/24/2020 | 31.3 | 33.7 | 29.1 | 3.6 | 2.3 |
| 7/1/2020 | 46 | 24.3 | 19.4 | 7.6 | 2.7 |
| 7/8/2020 | 36.2 | 31.4 | 23.8 | 5.9 | 2.7 |
| 7/16/2020 | 40.5 | 28.1 | 21.6 | 5 | 4.9 |
| 7/22/2020 | 38.1 | 37 | 19.9 | 2.3 | 2.7 |
| 7/29/2020 | 34.6 | 38.1 | 19.8 | 4.9 | 2.7 |
| 8/5/2020 | 37.8 | 30.2 | 19.8 | 8.5 | 3.6 |
| 8/12/2020 | 36.3 | 33.4 | 21.3 | 5.4 | 3.6 |
| 8/19/2020 | 38.5 | 32.7 | 21.6 | 3.6 | 3.6 |
| 8/26/2020 | 40.2 | 29.6 | 18.9 | 5.4 | 5.9 |
| 9/2/2020 | 40.9 | 28.3 | 21.9 | 5.8 | 3.1 |
| 9/9/2020 | 35.5 | 33.9 | 18.8 | 5 | 6.7 |
| 9/16/2020 | 34.6 | 33.3 | 24 | 5.4 | 2.7 |
| 9/23/2020 | 41.3 | 23.7 | 26.1 | 3.6 | 5.3 |
| 9/30/2020 | 41.3 | 29.3 | 18.8 | 6.3 | 4.4 |
| 10/7/2020 | 40.2 | 31.5 | 17.9 | 6.4 | 4 |
| 10/14/2020 | 36.8 | 33.3 | 20.1 | 6.7 | 3.1 |
| 10/21/2020 | 40.3 | 32.9 | 18.6 | 4.6 | 3.7 |
| 10/28/2020 | 44.3 | 27.4 | 17.1 | 5.3 | 5.9 |
| 11/4/2020 | 38.9 | 29.2 | 20 | 6.6 | 5.3 |
| 11/11/2020 | 43.2 | 34.2 | 14.9 | 4.9 | 2.7 |
| 11/18/2020 | 36.7 | 30.7 | 25.1 | 4.4 | 3.1 |
| 11/25/2020 | 39.8 | 31.7 | 20.8 | 3.6 | 4.1 |
| 12/2/2020 | 38.0 | 40 | 14.7 | 4.1 | 3.3 |
| 12/9/2020 | 43.0 | 28.2 | 15.1 | 5.7 | 8 |
| 12/16/2020 | 47.0 | 29.2 | 17.6 | 1.8 | 4.4 |
| 12/23/2020 | 50.1 | 26.3 | 19.1 | 3.6 | 0.9 |
| 12/30/2020 | 48.6 | 25.8 | 17.1 | 5.5 | 3.1 |
| 1/6/2021 | 38.9 | 35.1 | 17.1 | 7.1 | 1.7 |
| 1/13/2021 | 40.9 | 31.4 | 18.2 | 4.5 | 5 |
| 1/20/2021 | 40.3 | 28.7 | 19.9 | 6.4 | 4.6 |
| 1/27/2021 | 40.3 | 30 | 20.7 | 4.7 | 4.3 |
| 2/3/2021 | 40.9 | 31.1 | 21.4 | 2.6 | 4 |
| 2/10/2021 | 38.6 | 28.8 | 22.3 | 4.5 | 5.7 |
| 2/17/2021 | 34.6 | 30.4 | 22.7 | 6.7 | 5.7 |
| 2/24/2021 | 37.9 | 29.1 | 23 | 5.9 | 4 |
| 3/3/2021 | 39 | 27.6 | 21.4 | 6.7 | 5.2 |
| 3/10/2021 | 36.9 | 30.5 | 21.6 | 5.7 | 5.3 |
| 3/17/2021 | 36.3 | 28.1 | 24.9 | 7 | 3.6 |
| 3/24/2021 | 37 | 34.5 | 16.5 | 6 | 6 |
| 3/31/2021 | 39.4 | 26.2 | 23 | 6.7 | 4.7 |
| 4/7/2021 | 35 | 28 | 23.3 | 5.9 | 7.7 |
| 4/14/2021 | 32.2 | 29.5 | 24.5 | 6.9 | 7 |
| 4/21/2021 | 36.3 | 31.9 | 19.9 | 6.3 | 5.6 |
| 4/28/2021 | 35.7 | 27.6 | 21 | 8.7 | 7 |
| 5/5/2021 | 34.9 | 26.2 | 23.9 | 7.7 | 7.3 |
| 5/12/2021 | 33.4 | 27 | 22.4 | 9.7 | 7.6 |
| 5/19/2021 | 35.7 | 31 | 18.7 | 7.7 | 7 |
| 5/26/2021 | 25.1 | 33.7 | 23.9 | 9.3 | 8 |
| 6/2/2021 | 30 | 29 | 25.2 | 8 | 7.7 |
| 6/9/2021 | 26.9 | 25.9 | 29.7 | 7.4 | 10 |
| 6/16/2021 | 26.7 | 29.1 | 25.7 | 11.3 | 7.2 |
| 6/23/2021 | 26.7 | 22.5 | 31.4 | 12.8 | 6.7 |
| 6/30/2021 | 22.5 | 26.8 | 25.6 | 12.4 | 12.7 |
| 7/7/2021 | 28 | 28 | 25.9 | 11 | 7.1 |
| 7/14/2021 | 24.8 | 31.5 | 31.9 | 3 | 8.7 |
| 7/21/2021 | 24 | 30.3 | 25.6 | 10.8 | 9.3 |
| 7/28/2021 | 28.1 | 32.7 | 21 | 10 | 8.3 |
| 8/4/2021 | 27.5 | 30.9 | 25 | 8.3 | 8.3 |
| 8/11/2021 | 27.1 | 29.5 | 26.7 | 11 | 5.7 |
| 8/18/2021 | 24.3 | 28.2 | 28.9 | 9 | 9.5 |
| 8/27/2021 | 26.4 | 31.8 | 27.9 | 8.1 | 5.8 |
| 9/1/2021 | 30 | 31.3 | 22.7 | 9.4 | 6.7 |
| 9/8/2021 | 31.6 | 34.2 | 20 | 6 | 8.2 |
| 9/15/2021 | 25.9 | 30.8 | 32.4 | 6.6 | 4.4 |
| 9/22/2021 | 22.7 | 35.2 | 22 | 12.7 | 7.3 |
| 9/29/2021 | 21.2 | 30.6 | 24.6 | 17.3 | 6.3 |
| 10/6/2021 | 25 | 33.7 | 25 | 8.7 | 7.6 |
| 10/13/2021 | 22.9 | 27.3 | 29.3 | 11.3 | 9.2 |
| 10/20/2021 | 18.5 | 31.5 | 27.9 | 11.5 | 10.6 |
| 10/27/2021 | 23.2 | 27.1 | 27.2 | 14.4 | 8 |
| 11/3/2021 | 19.4 | 31.6 | 22.3 | 13.4 | 13.3 |
| 11/10/2021 | 19.3 | 24.3 | 24.1 | 18.8 | 13.6 |
| 11/17/2021 | 19 | 26.3 | 26.8 | 13.4 | 14.6 |
| 11/24/2021 | 19.8 | 29.8 | 32 | 11.3 | 7 |
| 12/1/2021 | 17.2 | 29.1 | 32.1 | 10.5 | 11.1 |
| 12/8/2021 | 20.6 | 25.4 | 27.5 | 11.7 | 14.8 |
| 12/15/2021 | 21.3 | 33 | 28.3 | 11.1 | 6.3 |
| 12/22/2021 | 20.3 | 30.8 | 26.8 | 13.2 | 9 |
| 12/30/2021 | 21.6 | 29.8 | 27.7 | 10.6 | 10.3 |
| 1/5/2022 | 22.6 | 29.1 | 29.3 | 11.7 | 7.3 |
| 1/12/2022 | 22.1 | 28.4 | 31.2 | 12.6 | 5.6 |
| 1/19/2022 | 21.5 | 33.8 | 25.2 | 12 | 7.5 |
| 1/26/2022 | 21.7 | 25.9 | 33.4 | 9.3 | 9.6 |
| 2/2/2022 | 17 | 27.1 | 32.7 | 11.3 | 11.9 |
| 2/10/2022 | 17.2 | 24.5 | 36.7 | 13.5 | 8.2 |
| 2/16/2022 | 19.9 | 25.3 | 30.5 | 13.8 | 10.6 |
| 2/23/2022 | 19.8 | 24.9 | 30.8 | 13.3 | 11.3 |
| 3/2/2022 | 14.4 | 27.8 | 32.2 | 14.2 | 11.4 |
| 3/9/2022 | 14.1 | 29.5 | 30.7 | 11.7 | 14 |
| 3/16/2022 | 14.6 | 29.8 | 26.4 | 14.9 | 14.3 |
| 3/23/2022 | 15.9 | 24.6 | 32.4 | 13.3 | 13.8 |
| 3/30/2022 | 13.3 | 26 | 26 | 16.7 | 18 |
| 4/6/2022 | 11.9 | 23.3 | 27 | 20 | 17.7 |
| 4/13/2022 | 15.1 | 22.6 | 24.7 | 18.9 | 18.7 |
| 4/20/2022 | 11.6 | 23.1 | 26.7 | 16.5 | 22 |
| 4/28/2022 | 16.7 | 23.4 | 28.9 | 14.2 | 16.8 |
| 5/4/2022 | 16.1 | 18.1 | 28.2 | 16 | 21.7 |
| 5/11/2022 | 15.6 | 20.4 | 25.6 | 19.1 | 19.3 |
| 5/18/2022 | 10.7 | 21.9 | 27.8 | 15.4 | 24.2 |
| 5/25/2022 | 8.3 | 20.8 | 31.3 | 15.3 | 24.3 |
