# Supplementary figures and images for: Checkpoint Travel Numbers as a Proxy Variable in Population-Based Studies During the COVID-19 Pandemic: Validation Study
Source: JMIR Public Health Surveill. 2023 Aug 29;9:e44950. doi: 10.2196/44950 (PMC10467631; doi:10.2196/44950)

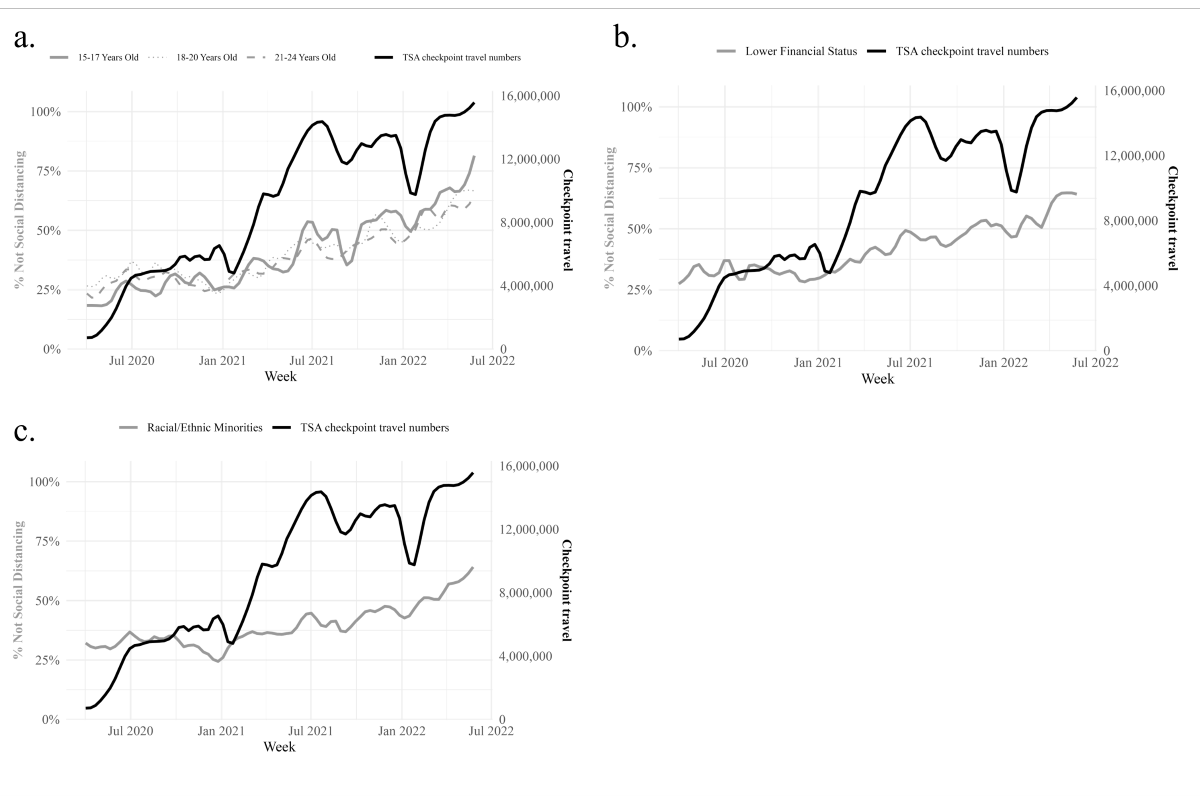

Supplement: Multimedia Appendix 3 [file publichealth_v9i1e44950_app3.png]

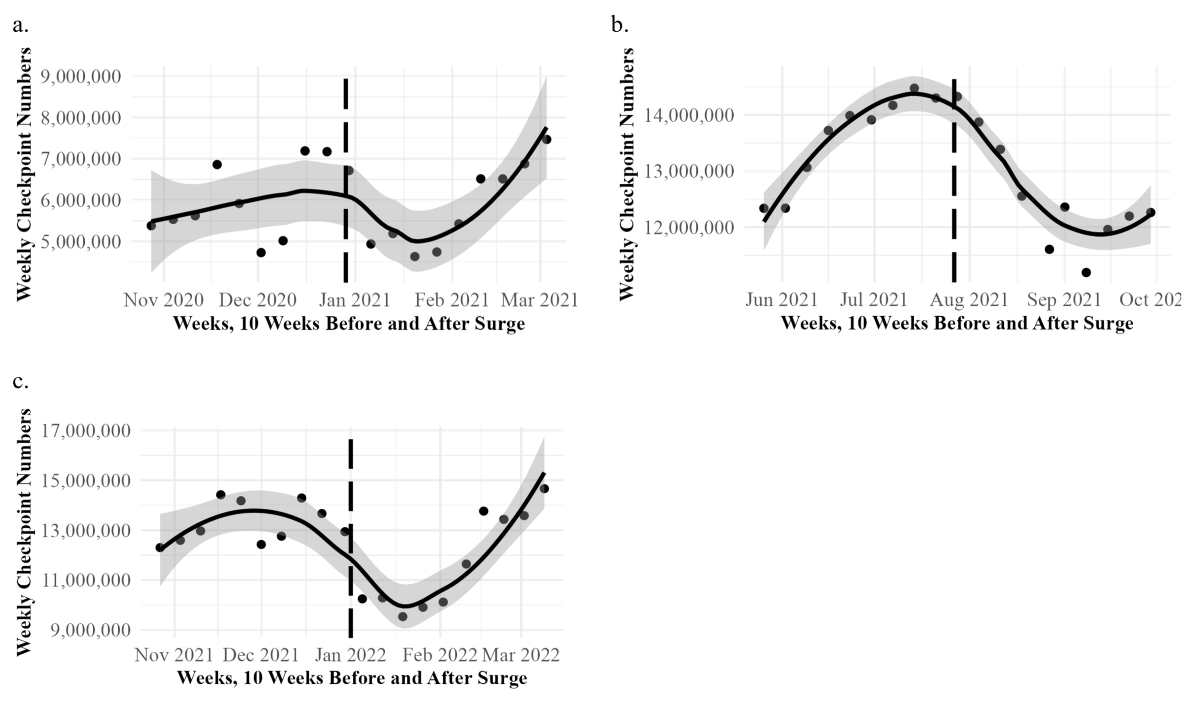

Supplement: Multimedia Appendix 4 [file publichealth_v9i1e44950_app4.png]
